# Risk and protective factors for transgender youths' substance use

**DOI:** 10.1016/j.pmedr.2019.100905

**Published:** 2019-05-22

**Authors:** Ryan J. Watson, Jaimie F. Veale, Allegra R. Gordon, Drew B.A. Clark, Elizabeth M. Saewyc

**Affiliations:** a University of Connecticut, United States of America; b University of Waikato, New Zealand; c Boston Children's Hospital, Harvard Medical School, United States of America; d University of British Columbia, Canada

**Keywords:** Transgender, Substance use, Protective factors, Probabilities

## Abstract

Research at the intersection of substance use and protective factors among transgender youth is scarce; emerging evidence suggests high risk for substance use for transgender youth. We analyzed data from 323 transgender youth aged 14–18 (*M*_age_ = 16.67) to investigate the extent that risk (enacted stigma) and protective factors (support from family, school, friends) were related to substance use (i.e., cannabis and tobacco use, binge drinking). Enacted stigma was linked to higher odds of substance use behaviors, family connectedness was related to lower levels of tobacco and cannabis use, and more than one protective factor significantly lowered the probability of engaging in substance use behaviors. Support from multiple sources may be differentially protective against substance use for transgender youth.

## Highlights

• Risk and protective factors are strongly related to substance use among transgender youth.
• Differing combinations illustrate the importance of social support in reducing substance use.
• High enacted stigma is linked to higher odds of substance use among transgender youth.
• More than one protective factor lowers the probability of engaging in substance use.

## 1 Introduction

Though the prevalence of substance use has declined among youth in the past decade, there is evidence that the declines are not the same among sexual and gender minority youth (e.g., lesbian, gay, bisexual, queer, and transgender) (Hughes and Eliason, 2002; Ramirez-Valles et al., 2008), and some evidence that health disparities may be widening (Homma et al., 2016; Watson et al., 2017a). Levels of substance use (Garofalo et al., 2006; Russell et al., 2011) among gender minority youth are disproportionately high. For example, a recent study that utilized representative data from California found stark disparities in substance use for transgender youth, such that they were at 2.5–4 times higher risk of substance use (depending on the substance) compared to cisgender youth (Day et al., 2017). Not all substance use is problematic (e.g., contributing to individually experienced harms) (Asbridge et al., 2014), however tobacco and alcohol continue to be leading causes of premature death and chronic health problems (Peterson et al., 1980). According to recent global review of the transgender health literature, little scholarly attention has focused on substance use and its link to risk and protective factors for transgender youth (Reisner et al., 2016).

High rates of substance use have been consistently documented among transgender youth. In one study conducted in San Francisco, California among 292 transgender girls/women aged 16–24, scholars found that 69% reported use of at least one type of drug (excluding alcohol) in the past six months. Additionally, half of the sample (51%) reported binge drinking in the past six months (Rowe et al., 2015). While most studies focus on alcohol, marijuana, and cigarette smoking, there is evidence that smaller, yet still significant, numbers of transgender youth use non-marijuana illicit drugs (e.g., 19% reported non-marijuana illicit drug use in a Massachusetts sample of transgender youth) (Keuroghlian et al., 2015). Other studies with comparison groups confirm higher rates for gender minority populations: using US data, Reisner and colleagues (Reisner et al., 2015) found that almost half of transgender or gender nonconforming youth reported past-year drinking of alcohol, compared to about 38% of cisgender boys and girls (a statistically significant difference), and that transgender and gender nonconforming youth were more likely to have *ever* smoked cigarettes (28.6%) and cannabis (27.7%) compared to their cisgender counterparts (about 20% and 12.5%, respectively). Similar to research from a decade ago conducted in Chicago, Illinois (Garofalo et al., 2006), Veale et al. (2015) found that substance use rates were high in a 2014 Canadian sample: 49% of youth aged 19–25 reported smoking cigarettes and 69% reported using marijuana in their lifetime. In a sample of 28,662 adult participants in Massachusetts, scholars found that transgender adults had nearly 3 times the odds of smoking compared to cisgender adults (Conron et al., 2012).

Enacted stigma includes discrimination, harassment, and violence experienced by individuals – in particular, gender minority populations (Lyons et al., 2015). Enacted stigma has previously been correlated with early withdrawal from treatment programs among transgender adults (Lyons et al., 2015). Research suggests that in part because of enacted stigma, transgender individuals are oftentimes more likely to use substances compared to their cisgender counterparts (Coulter et al., 2015; Newcomb et al., 2014). For example, in a three-year prospective study among 230 transgender adult women living in New York, gender-related stigma and stress was significantly linked to using more substances (Nuttbrock et al., 2014). In this study, we conceptualized enacted stigma as a risk factor that may potentially increase the likelihood of problematic substance use by transgender young people, who may use substances as a coping mechanism against enacted stigma and the stress associated with this stigma.

Recently, scholars have considered protective factors that might intervene to buffer stigma and prevent negative health outcomes within transgender populations. A limited amount of previous research has explored how the presence of protective factors mitigates a number of negative outcomes. For example, Klein and Golub (Klein and Golub, 2016) found that among 3458 transgender and gender nonconforming adults in the US National Transgender Discrimination Survey, family rejection was related to suicidality and higher substance use after controlling for a number of factors such as age, race, sex assigned at birth, and gender identity. Additionally, Canadian transgender youth aged 16–24 with supportive parents reported lower rates of depressive symptoms, suicide contemplation, and suicide attempt than youth with families that are somewhat to not at all supportive (Veale et al., 2017). Higher levels of family connectedness have also been linked to better self-reported mental health by 14–18 year-old Canadian transgender youth (Travers et al., 2012).

In addition to family, scholars have conceptualized supportiveness of friends and schools as two important social factors that are linked to lower rates of potential health-compromising behaviors (Watson et al., 2019). For example, using data from Massachusetts, scholars found that friend support significantly decreased the likelihood of fasting and using diet pills/laxatives to lose weight (Watson et al., 2017b). In the Canadian Transgender Youth Health Survey, 79% of the 923 transgender youth reported asking a friend for help, and 84% of the youth reported that their friends were helpful in giving the needed support (Veale et al., 2015). Similarly, higher levels of school connectedness have been linked to better mental health for transgender youth (Veale et al., 2015), such that youth with higher feelings of connectedness to school were also more likely to report good or excellent mental health compared to their counterparts with lower school connectedness.

There is a lack of research on how protective and risk factors relate to substance use among transgender youth. If left unexplored, opportunities for meaningful advances in interventions for this population may be missed. In this paper, we consider three specific protective factors that have been found to be protective for the general youth population (Klein and Golub, 2016; Veale et al., 2017; Travers et al., 2012), and may likewise be relevant for transgender youth: family connectedness, caring friends, and school connectedness. We also consider various combinations of protective factors and enacted stigma, to uncover the relations between these factors and the use of alcohol, tobacco, and cannabis among transgender young people.

## 2 Method

### 2.1 Sample

Data were drawn from the Canadian Trans Youth Health Survey, which was conducted online from October 2013 to May 2014 in both English and French. The survey was open to youth aged 14–25 (mean age = 20, *SD* = 3.03) who self-identified as transgender. We recruited a total of 923 participants via emails distributed to community-based organizations and health professionals who work with transgender youth, Facebook advertisements, and our networks of transgender youth advisory council members. More information about the sample can be found elsewhere (Veale et al., n.d.; Veale et al., 2017). Ethics approval was received from several university research ethics boards in Canada.

We used a subset of data from younger youth aged 14–18 (*n* = 323, mean age = 16.67, *SD* = 1.18) because the long-term health implications of early onset substance use warrant an examination of the relationships among high rates of substance use and risk/protective factors for this population. In addition, substance use behaviors may be more normalized (or legal, in the case of alcohol) for older transgender youth. More than three quarters of younger youth spoke only English at home (80%) and the majority was Canada-born (88%). Just under three quarters (70%) identified as White only and 15% of the sample identified as Indigenous or Aboriginal.

We asked several questions related to transgender identity. For this analysis, the item used to assess gender identity was: “When a person's sex and gender do not match, they might think of themselves as transgender. Sex is what a person is born. Gender is how a person feels. Which one response best describes you?” with response options, “I am not transgender,” “I am transgender and identify as a boy or man,” “I am transgender and identify as a girl or woman,” and “I am transgender and identify in some other way.” This item was used to categorize participants as transgender girls/women, transgender boys/men, and non-binary. Among the 323 youth, 140 identified as transgender boys/men (47%), 32 as transgender girls/women (11%), and 128 as non-binary youth (42%). Of the 128 non-binary youth, 18 were assigned male at birth and 110 were assigned female at birth. The other 23 youth did not indicate their specific gender identity other than a “transgender” identity.

### 2.2 Measures

#### 2.2.1 Enacted stigma

We created an Enacted Stigma Index based on previous research (Poon et al., 2012) through a sum score of stigma experiences reported by participants; these 29 items included harassment, bullying, discrimination, and violence (see Table 1). Most items asked whether a stigma experience occurred, and a few asked participants to respond with a frequency, which were then dichotomized to create yes/no responses. Each of the items was scored 0 or 1, and thus the index was coded from 0 to 29. The mean score for this index (i.e., average number of enacted stigma experiences reported) was 11.75 (*SD* = 6.55, median = 12.0).

**Table 1:** Enacted stigma experience items (total possible score, 0–29).

1. Number of reasons for experiencing discrimination (past year; scored 0–11) for 11 different reasons: ethnicity or culture, race or color, physical appearance, religion, sexual orientation, age, disability, language, gender identity, or other reason
2. Number of reasons for experiencing verbal harassment for: (past year; scored 0–4)
a. race or culture
b. sexual orientation
c. body size/shape/appearance
d. gender identity
3. Been bullied on the internet (ever)
4. Bullying (past year; scored 0–2)
a. been bullied/taunted/ridiculed
b. been bullied at school, including being repeatedly teased, threatened, hit, kicked, or excluded by another student or group of students
5. Physically threatened/injured (past year)
6. Threatened with weapon (past year)
7. Physically hurt by someone in family (past year)
8. Sexual abuse (ever)
9. Sexual touch by older or stronger family member (ever)
10. Unwanted sexual touch by any adult or person outside family (ever)
11. Physically hurt or forced sex by a date (ever)
12. Physically forced into sexual intercourse (ever)
13. Sexual harassment (past year; scored 0–2):
a. unwanted sexual comments
b. unwanted sexual touch
14. Sexual exploitation, i.e., traded sexual activity for money, food, shelter, drugs/alcohol (ever)

#### 2.2.2 School connectedness

We used a 5-item scale to measure school connectedness (McNeely, 2006; Resnick et al., 1997). The scale measured feelings of belonging, engagement, and connection to one's school (α = 0.87). Response options ranged from 1 (*strongly disagree*) to 5 (*strongly agree*). As an example, one question asked, “*I feel I am part of my school*”. This scale has been tested for reliability and measurement stability across 18 ethnic groups (Furlong et al., 2011) as well as among sexual minority youth (Saewyc et al., 2009).

#### 2.2.3 Family connectedness

A 7-item scale was used to assess family connectedness (α = 0.92,). The scale measured the degree to which youth felt close and connected with their mother, father, and family (Resnick et al., 1997; Saewyc and Edinburgh, 2010). As an example, one question asked, “*How much do you feel that your family cares about your feelings?*”. Response options ranged from 1 (*not at all*) to 5 (*very much*).

#### 2.2.4 Perception of friends caring

To assess the participants' perceptions that their friends cared about them, we used one item (Watson et al., 2017b) that asked participants, *how much do you feel that your friends care about you?* Response options ranged from 1 (*not at all*) to 5 (*very much*).

#### 2.2.5 Substance use

We asked participants, *How many times in the last 4 weeks have you had 5 or more drinks of alcohol on the same occasion?* With an 8-point response scale from *never drank alcohol in my lifetime* to *5 or more times*. We also asked participants, *In the last 4 weeks, how often (if ever) did you smoke a cigarette?*, and *In the last 4 weeks, how often (if ever) did you use cannabis (also known as marijuana, weed, pot, grass, hashish, hash, hash oil)?* with a 7-point response scale for both questions, from *did not ever use in the past 4 weeks* to *more than once each day*. We dichotomized the data for these three questions into categories of use in the last month and no use in the last month.

### 2.3 Statistical analyses

All analyses were conducted with SPSS version 22. All models were adjusted for age given there were several significant associations found between age and substance use outcomes. Other demographic variables (e.g., race) were not significantly associated with study outcomes; thus, we did not include these variables in our final models. Missing data for study outcome variables ranged from 24% to 31%, due mainly to participant attrition due to the entire survey taking most participants over 30 min to complete.

We utilized bivariate and multivariate logistic regression models to test associations between risk and protective factors and substance use. We used the resulting odds ratios from these models to produce probability profiles, which are used to *illustrate* the predicted probability of a transgender youth in our sample reporting a substance use outcome given high (90th percentile) or low (10th percentile) levels of enacted stigma. Probability profiling provides insight into how patterns of co-occurring risk (e.g., enacted stigma) and protective (e.g., family support) factors are related to outcomes (e.g., substance use). Examples of probability profiling can be found from a variety of scholarly works (Rubenstein et al., 1989; Pettingell et al., 2008; Resnick et al., 2004). Below we describe the steps to produce probability profiles.

To begin our statistical analyses, we first compared all protective factors on a common metric (by standardizing the scales from 0 to 1) so that the relative strength of the effects on each substance use outcome were interpretable. We used bivariate logistic regression models to test whether each single risk and protective factor was significantly associated with substance use behaviors. We then included age as well as all the risk and protective factors that had significant bivariate associations into a multivariate logistic regression model for each substance use outcome.

The parameter estimates for the risk and protective factors from these models were then used to calculate probability profiles using Microsoft Excel. These profiles illustrated different combinations of risk and protective factors in relation to risk of a participant engaging in substance use. Specifically, this method uses different combinations of “low” (10th percentile) and “high” (90th percentile) levels of risk and protective factors to calculate the risk of a participant engaging in substance use behaviors, given those different levels of risk and protective factors.

## 3 Results

Among our sample, 24.2% of transgender youth reported past-month cannabis use, 23.4% reported past-month cigarette smoking, and 19.5% reported past-month binge drinking. Table 2 displays the bivariate and multivariate models for the three substance use outcomes. Two different protective factors were significantly related to cannabis and cigarette outcomes, although the statistically significant effects disappeared after including them in age-adjusted multivariate models. As expected, for each of the outcomes, higher numbers of enacted stigma experiences were positively associated with odds of substance use, while protective factors were mostly negatively related to these behaviors, with the exception of perception of friends caring for binge drinking. Odds ratios for the enacted stigma index correspond to the calculated odds of reporting substance use in relation to a one-point increase on the enacted stigma index (i.e., one experience of enacted stigma). For example, each additional one experience on the enacted stigma index corresponded to an 11% increase in the odds of past month cannabis use among transgender youth.

**Table 2:** Prevalence of substance use bivariate and multivariate logistic regression models.

	Bivariate model	Multivariate model
	Odds ratio (95% CIs)	Odds ratio (95% CIs)
Past month smoking cannabis	*N* (yes) = 54, *N* (no) = 169	
Enacted Stigma Index	1.11 (1.02–1.21)⁎⁎	1.09 (0.99–1.22)
Family Connectedness Scale	0.12 (0.03–0.46)⁎⁎	0.46 (0.09–2.40)
School Connectedness Scale	0.45 (0.12–1.67)	Not included
Perception of friends caring	0.29 (0.10–0.83)⁎⁎	0.35 (0.10–1.21)
Age	–	0.98 (0.72–1.34)
Past month smoking cigarettes	*N* (yes) = 58, *N* (no) = 189	
Enacted Stigma Index	1.12 (1.03–1.21)⁎⁎	1.08 (0.99–1.19)
Family Connectedness Scale	0.06 (0.01–0.55)⁎	0.10 (0.01–1.51)
School Connectedness Scale	0.10 (0.01–0.75)⁎	0.41 (0.04–4.39)
Perception of friends caring	0.56 (0.12–2.73)	Not included
Age	–	1.27 (0.93–2.03)
Past month binge drinking	*N* (yes) = 44, *N* (no) = 182	
Enacted Stigma Index	1.11 (1.02–1.12)⁎⁎	–
Family Connectedness Scale	0.51 (0.12–2.06)	–
School Connectedness Scale	0.68 (0.17–2.78)	–
Perception of friends caring	1.39 (0.43–4.56)	–

⁎ p < .05.

⁎⁎ p < .01.

Odds ratios for the protective factors correspond to the calculated odds of those who scored at the highest possible score for the scale versus those at the lowest possible score. For example, youth who scored the highest on the Family Connectedness Scale were about 88% less likely (*OR* = 0.12) to report past-month smoking of cannabis compared to their counterparts who scored the lowest on this item. Because no protective factors were significantly associated with past month binge drinking, we did not include this outcome in the multivariate models and probability profiles. However, higher numbers of types of enacted stigma were associated with increased odds of binge drinking for transgender youth.

**Fig. 1:**
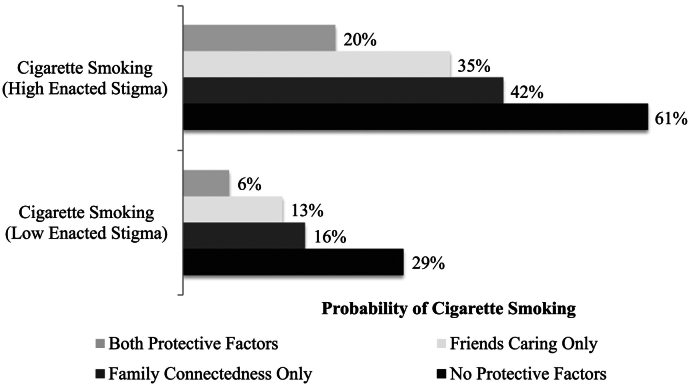
Probability of past-month cigarette smoking by level of enacted stigma.

**Fig. 2:**
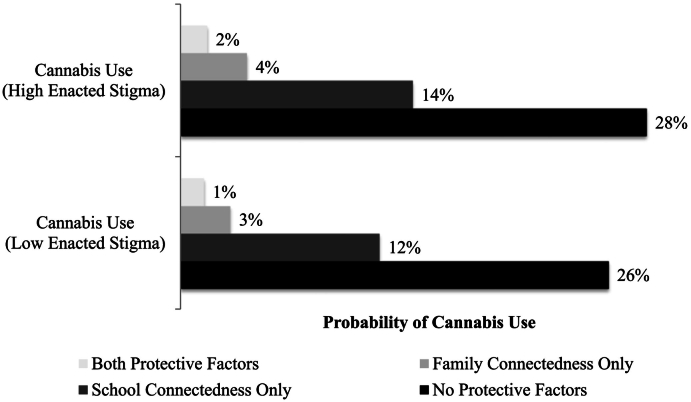
Probability of past-month cannabis use by level of enacted stigma.

Probability profiles estimated for combinations of risk and protective factors for cigarettes or cannabis use are presented in Fig. 1, Fig. 2. Youth who reported both high levels (90th percentile) of enacted stigma and low levels (10th percentile) of protective factors had the greatest probabilities of engaging in substance use. Youth who reported high levels of two protective factors (combinations of two) had lower probabilities of substance use than youth with one or no protective factors. Fig. 1 displays the probability profiles of past-month cigarette use for transgender youth, and Fig. 2 displays the probability profiles of past-month cannabis use. These Figures illustrate the findings from the multivariate models, in which the previously statistically significant effects (protective factors' relations with substance use outcomes) were no longer statistically significant when including other protective factors and age in the multivariate models.

## 4 Discussion

We found high rates of substance use for transgender youth; about a quarter of youth in our sample reported smoking cigarettes or cannabis in the past month. Fewer youth reported past-month binge drinking (19.4%). These findings are in line with previous research that found similar prevalence of cigarette and cannabis use among transgender youth (Day et al., 2017; Reisner et al., 2016; Reisner et al., 2015); although *ever* use of cigarettes and cannabis have been measured in previous studies, while we studied past-month use. Additionally, Reisner and colleagues found lower rates than we did for *regular substance use* (17.4% and 14.8% for cigarettes and cannabis, respectively) (Reisner et al., 2015).

Interestingly, many of the statistically significant associations between protective factors and substance use outcomes were no longer statistically significant in our multivariate models. These null results could be due to a lack of statistical power (i.e., low prevalence of substance use behaviors combined). Additionally, we observed no associations between binge drinking and the three protective factors examined. It is plausible that alternate types of protective factors not captured here might play a role. For example, neighborhood level factors such as community cohesion have been found to buffer against binge drinking in youth and young adult populations (Cleveland et al., 2008). Because binge drinking is often a social activity, it may be that the beneficial effects of social support in buffering against binge drinking are less present in the lives of trans youth, who may be socially isolated due to their gender, or that these effects are outweighed by the function of binge drinking as a means to cope with gender-related distress. Additional risk factors (e.g., isolation) and protective factors (e.g., connection to transgender communities) against binge drinking and other substance use among transgender youth should also be explored in future studies.

Our findings support prior research demonstrating that exposure to stigma and violence is associated with elevated risk of substance use among transgender youth (Rowe et al., 2015; Reisner et al., 2015; Coulter et al., 2015) and adults (Nuttbrock et al., 2014). Transgender youth who experienced high levels of enacted stigma had a greater probability of engaging in past month cigarette smoking, cannabis use, and binge drinking compared to those with low enacted stigma experiences.

### 4.1 Limitations

Our findings should be considered in light of several limitations. First, our focus on the most common substances means that our findings may not apply to experiences with other drugs, such as cocaine or opioids. We did not include these experiences in this paper due to a low prevalence of these experiences among our sample would not have allowed us to examine risk and protective factors. Second, due to sample size limitations, we were not able to explore potential differences in substance use risk and protective factors by gender identity, and many significant findings from the bivariate models were no longer statistically significant in multivariate models. Given that gendered patterns of substance use have been observed in cisgender youth samples (Newcomb et al., 2014), future research could explore such relationships in larger samples of transgender youth. Third, we were missing substance use data on a sizable number of transgender participants due to our questions on substance use appearing near the end of a long questionnaire; though we do not believe this data was systematically missing, we acknowledge the limitations associated with missing data. Additionally, this study was based on a non-probability sample, and may not be representative of the general population. However, the transgender youth population has recently been estimated to be about 1% (Clark et al., 2014), and thus population-based sampling would require an extremely large sample in order to be able to conduct an in-depth study of risk and protective factors.

### 4.2 Implications

This study offers insights into the role of risk and protective factors related to substance use among transgender youth, with several clinical and public health implications. This contribution is important in light of the growing evidence that transgender populations are at elevated risk of substance use and adverse mental health outcomes compared to their cisgender peers (Reisner et al., 2016; Reisner et al., 2015). Our findings suggest a need to integrate an understanding of multi-level risk factors for substance use among transgender youth into policy development, program design, and service delivery. Recognition of the impacts of societal stigma is essential for the development of transgender culturally competent services (Cook et al., 2014; Hughto et al., 2015).

Although not statistically significant in our multivariate models, our univariate models and our best estimates of predicted probabilities also illuminated important associations between protective factors and reduced probability of recent tobacco and cannabis use. Youth may engage in problematic substance use to cope with enacted stigma, however, this substance use may be preventable through environmental change (e.g., reduced enacted stigma, increased supports). Even youth with high enacted stigma experiences had markedly lower probabilities of cigarette and cannabis use if they had at least two protective factors, compared to one or no protective factors. This raises research questions about how a variety of protective resources may beneficially cluster and build on each other over time.

### 4.3 Future directions

Going forward, there is a need to conduct both population-based surveillance surveys that include questions about gender identity, as well as non-probability samples focused on transgender youth, such as the present survey; this will allow for both generalizable prevalence estimates and in-depth analyses of mechanisms to reduce risk and increase well-being among transgender youth. Further exploration of whether some forms of substance use may function as a factor protecting youth from higher-risk health behaviors, as well as research focused on harm reduction strategies to minimize negative effects of problematic substance use (Asbridge et al., 2014) and differentiation of problematic and non-problematic substance use are also warranted (Thompson et al., 2018). Stakeholders can apply these findings to inform substance use intervention programs. Supportive, gender-affirming substance use treatment programs have been linked to positive experiences among adult transgender clients (Lyons et al., 2015), however research with transgender youth indicates that “ironically, the addictions treatment settings and service providers tended to both mirror and reinforce the conditions that contributed to substance abuse in the first instance (p. 372)” (Klein and Golub, 2016). Exploration of how transgender culturally safer services – characterized by transgender-inclusive policies and procedures, staff training, and supportive, gender-affirming treatment milieus (Nuttbrock, 2012)—may increase representation in treatment and serve as a protective factor among transgender youth populations is also worthy of future investigation. Last, clinicians should encourage family support for transgender youth, and can advocate for inclusive school policies. Future research with transgender youth and families that uses longitudinal designs to identify specific mechanisms buffering against substance use over time is also needed.

## Declaration of Competing Interest

All authors disclose no potential conflicts, real and perceived.
